# Mycotoxins as Emerging Contaminants. Introduction to the Special Issue “Rapid Detection of Mycotoxin Contamination”

**DOI:** 10.3390/toxins13070475

**Published:** 2021-07-09

**Authors:** András Székács

**Affiliations:** Agro-Environmental Research Centre, Institute of Environmental Sciences, Hungarian University of Agriculture and Life Sciences, Herman O. út 15, H-1022 Budapest, Hungary; szekacs.andras@uni-mate.hu

Concerns for human and environmental health regarding mycotoxins are predominantly raised in connection with their occurrence in food and feed (especially in grains) [1,2]. Thus, mycotoxin contamination is an emerging problem in agriculture. These toxic secondary metabolites produced by some fungal species belong to chemically diverse groups of low molecular weight fungal metabolites with a range of toxic effects including genotoxicity and endocrine disruption [3,4,5,6]. In addition, mycotoxins have been identified recently as emerging contaminants in aqueous environments as well [7,8]. In turn, rapid detection of mycotoxins became an essential requirement in both food/feed and environmental monitoring that also triggered method development [9,10].

Recent developments, utilization, evaluation and possible improvements of methods that allow rapid, sensitive, and accurate detection of various mycotoxins have been chosen to be the topic of this Special Issue. Overall, 56 authors contributed 13 articles (12 original research articles and a review) discussing various aspects of mycotoxin research, but with mycotoxin analysis involved in all cases. Thus, through a compilation of current progress in the field, the Special Issue focuses on several aspects of mycotoxin analysis. Its scope encompasses classical instrumental analytical or biosensoric method development, sample preparation and handling to support method accuracy, as well as applications in routine monitoring or in decontamination assessment.

## 1. Method Development

Panasiuk et al. [11] developed an ultra-performance liquid chromatography-tandem mass spectrometry (UHPLC-MS/MS) method for a range of target mycotoxins including deoxynivalenol (DON), 3- and 15-acetyl-DON, DON-3-glucoside, nivalenol (NIV), and fusarenone-X. Sample preparation for the method included solid–liquid extraction, dispersive solid-phase extraction (QuEChERS), solid-phase extraction with hydrophilic-lipophilic balance column, and several immunoaffinity columns; the highest efficacy being achieved with the last. However, of the six immunoaffinity columns tested, none showed cross-reactivity to all of the mycotoxins, therefore no single immunoaffinity separation can be advised. The optimized method using a Mycosep 225 Trich column clean-up was validated with a large number of feedstuff samples including wheat, maize, and animal feeds. A similar LC-MS/MS-based procedure is reported by Nakhjavan et al. [12] for multi-mycotoxin analyses. The method employing immunoaffinity clean-up, solid-phase extraction, or QuEChERS sample preparation was optimized for simultaneous quantitation of aflatoxins (aflatoxins B1, B2, G1 and G2, AFB1, AFB2, AFG1, and AFG2), ochratoxin A (OTA), zearalenone (ZEN), deoxynivalenol (DON), NIV, diacetoxyscirpenol, fumonisins (fumonisins B1, B2 and B3, FB1, FB2, and FB3), T-2 toxin and HT-2 toxin in feed, and it allows limits of detection (LODs) ranging between 0.0003 and 0.05 µg/mL for the various mycotoxins tested.

Majdinasab et al. [13] reviewed colorimetric methods for industrial monitoring of mycotoxins in food and feed e.g., grains and cereals, grape juice, or red wine, and discuss the advantages and disadvantages for each method. Colorimetric strategies for various mycotoxins including T-2, DON, OTA, aflatoxins, ZEN, or FB1 (but not to FB2 or FB3) consist of enzyme-linked assays, lateral flow assays, microfluidic devices, and homogenous in-solution strategies that can utilize various (bio)receptors such as antibodies or aptamers.

The development of several immunoanalytical methods for mycotoxin detection is presented in the Special Issue. A competitive nanoparticle-based magnetic immunodetection assay for the detection and quantification of AFB1 with a LOD of 1.1 ng/mL is reported by Pietschmann et al. [14]. The method is based on magnetic separation of streptavidin-labeled magnetic particles, using an immobilized AFB1 antigen and biotinylated monoclonal AFB1-specific antibodies. The binding of antibodies to the immobilized antigen is competed by the free analyte (AFB1) in the solution (sample). Bound (i.e., uninhibited) antibodies on the solid surface are detected by frequency mixing magnetic detection. The LOD of the method is 1.1 ng/mL, comparable to a laboratory-based enzyme-linked immunosorbent assay (ELISA) method with a LOD of 0.29–0.39 ng/mL. The development of a portable instrument for ZEN by enzyme-linked fluorescent immunoassay (ELFIA) is reported by Gémes et al. [15], but as this instrument is a novel application for detection of mycotoxins as emerging water contaminants, it is discussed among the applications of routine monitoring (see Section 3. Applications in routine monitoring).

Several immunosensors on the basis of the same ZEN-specific polyclonal antibody are presented in the Special Issue for the detection of ZEN. An immobilized antibody-based competitive optical planar waveguide-based immunosensor by Nabok et al. [16] allowed a concentration-dependent detection of ZEN in the 0.01–1000 ng/mL concentration range. The optimized experimental benchtop planar waveguide setup is planned to be further developed into a portable hand-held biosensor including the signal processing electronics, suitable for in-field use. Using a similar sensor design but utilizing both immobilized antibody- (direct) and immobilized antigen-based (competitive) architectures, novel optical waveguide light mode spectroscopy (OWLS)-based immunosensors are reported by Székács et al. [17]. Covalent immobilization on the sensor surface was devised by epoxy-, amino-, and carboxyl-functionalization, and standard sigmoid curves in the optimized sensor formats allowed an outstanding LOD of 0.002 pg/mL for ZEN in the competitive immunosensor setup with a dynamic detection range of 0.01–1 pg/mL ZEN concentrations. The OWLS format represents five orders of magnitude improvement in LOD compared to the corresponding competitive ELISA, and the selectivity of the immunosensor for ZEN is outstanding on the basis of cross-reactivities determined for structurally related and unrelated compounds. The method was shown applicable in maize extract.

In addition to immunoanalytical (antibody-based) setups, the development of a label-free aptamer-based fluorescent sensor is reported by Qian et al. [18] for the detection of OTA. The aptasensor utilizing a nucleotide recombination hybridization chain reaction amplification element allows high selectivity for OTA with a LOD of 2.0 pg/mL (4.9 pM). The elegant aptamer setup utilizes two hairpin nucleotide probes (H1 and H2). H1 contains a central loop portion capable of specific complex formation with OTA and two 6-nucleotide long terminal sequences complementary with each other. H2 is similar in structure, where the central loop is a G-quadruplex sequence capable to bind with N-methyl-mesoporphyrin IX and thus, forms a complex with enhanced fluorescent excitability. In the system, complex formation between OTA and H1 initiates repeated recombination-driven binding of numerous H2 probes, each incorporating N-methyl-mesoporphyrin IX molecules into the elongating H2 chain and resulting in amplification of the fluorescent signal. Other mycotoxins (ochratoxin B, AFB1) do not cross-react with the detection system and do not disturb the binding of OTA either. The detection method was demonstrated to be effective in wheat flour and red wine as commodity matrices.

## 2. Sample Preparation and Handling to Support Method Accuracy

As seen also from studies on method development in this Special Issue [11,12], sample preparation is a step of key importance in the chemical analysis process; not only due to its required features of applicability and recovery, but also because novel standardized methods, such as the QuEChERS dispersive solid-phase extraction protocol can facilitate standardization of the analytical procedure improving inter-laboratory standard errors. The work of Kibugu et al. [19] clearly illustrates the importance of appropriate sample selection and preparation methods to maintain analysis performance quality descriptors including accuracy, precision, linearity, robustness, and ruggedness, as well as limits of detection and quantification. Their detailed statistical analysis of the determination of AFB1 content in chicken feed, using hierarchical sampling (from primary to quaternary with gradually decreasing sample sizes), wet milling with solvent extraction, and AFB1 quantification by a commercial ELISA kit, indicates accurate, precise, stable, reliable, and cost-effective analysis with improved inherent variability, which allows the processing of a lowered recommended test portion sample size of 50 g, and is suitable for laboratories not equipped with automated sample-splitting equipment.

## 3. Applications in Routine Monitoring

Analytical approaches utilized in practical applications may not have to be entirely based on novel principles—application of traditional detection methods can be devised for given tasks. The work reported by Alshannaq et al. [20] adapts a high-performance liquid chromatography method coupled with diode array (DAD) and fluorescence (FLD) detectors to screen for the possible presence of aflatoxins (AFB1, AFB2, AFG1, AFG2) in aflatoxigenic and non-toxigenic laboratory fungal cultures of *Aspergilli*, including *Aspergillus flavus*, *A. oryzae*, and *A. parasiticus*. In their method, readily available and easily applied in most mycology laboratories, the limit of quantification (LOQ) for AFs was found to be 2.5 to 5.0 ng/mL with DAD and 0.025 to 2.5 ng/mL with FLD with medium recoveries of 76–88%. Hong et al. [21] apply an immunochromatographic assay based on digital detection using colloidal gold nanoparticles labeled to monoclonal antibodies to detect ZEN in authentic cereal (corn, wheat, wheat flour, cereal product) and feed samples within a monitoring campaign carried out in China in 2019. Their survey included 187 cereal and cereal product samples and allowed a LOD of 0.25 ng/mL and recoveries between 87 and 117%.

A possible route for mycotoxin exposure has been linked to mycotoxins as surface water contaminants [7,8,22,23]. The occasional occurrence of mycotoxins in surface and drinking water is not a newly identified phenomenon, but its particular significance has been emphasized lately [7,8,22,24,25,26], classifying mycotoxins and their metabolites as emerging surface water contaminants [7,27], and assessing their routes of occurrence [28,29]. Gémes et al. [15] report the development of an ELFIA method as a module of a portable, in situ fluorimeter instrument installed in a mobile laboratory vehicle to detect ZEN in water with a LOD of 0.09 ng/mL. This LOD appears to be quite favorable compared to reported ELISAs, but a major advantage of the ELFIA method lies in its combined in situ applicability in the determination of important water quality parameters detectable by induced fluorimetry—e.g., total organic carbon content, algal density or the level of other organic micropollutants. The immunofluorescence module also appears to be flexible; with the use of other expedient antibodies it can be expanded to other target analytes.

Mycotoxins are also emerging contaminants in traditional matrices (commodities, feedstuff) in previously atypical geographical areas due to pathogen migration caused by climate change [30,31,32,33]. In consequence, decontamination by the use of suitably isolated metabolic enzymes capable to decompose, preferably selectively, certain mycotoxins is of great interest both from the aspects of fundamental research and technology development. Thus, enzymatic decomposition [34] and surface binding on microbial cell walls [35,36] of mycotoxins have been extensively studied, and two studies have been devoted to this topic in this Special Issue by Kosztik et al. [37] and Bata-Vidács et al. [38]. By their cell wall polysaccharides binding various mycotoxins, certain microbes are capable of absorbing, or in rare cases degrading, these substances. Thus, these microorganisms can be utilized in the biological detoxification of given mycotoxins. Such binding potential of *Lactobacilli* [37] and non-*Lactobacillus* lactic acid bacteria [38] towards AFB1 and sterigmatocystin (ST) is reported in this Special Issue, as the first report on microbial ST binding. Among 105 phylogenetically characterized *Lactobacillus* strains, 14 strains were able to bind AFB1 above 5%, 58 strains showed minor (below 3%) binding capacity, and 33 strains could not bind the mycotoxin. The highest AFB1 binding capacities (8–12%) were obtained for a strain of *L. pentosus* and three strains of *L. plantarum*. In addition, among 49 lactic acid bacteria other than lactobacilli, three strains of *Pediococcus acidilactici*, as well as one strain of *Enterococcus hirae*, and one of *E. lactis* had higher AFB1 binding ability (7.6%, 4.6%, 4.6%, 4.6%, 3.5%, respectively). Among 39 similarly phylogenetically characterized *Lactobacillus* strains, 27 and 12 strains were able to bind ST above 5% and between 0.8% and 5%, respectively. The highest ST binding capacities (above 20%) were obtained for five strains of *L. plantarum*, a strain of *L. paracasei*, and a strain of *L. pentosus*. In addition, the ST binding ability of strains belonging to the genus *Pediococcus* was found to be 2–3 times higher than the AFB1 binding capacities. The best AFB1 binding *Pediococcus* strain was also the best ST binding. This can be explained by the fact that the two structurally similar mycotoxins bind to the same cell wall polysaccharide receptor of the bacterium.
